# The visualization of low-molecule phenol (LMP) and copper naphthenate on treated wood using X-ray microtomography

**DOI:** 10.1038/s41598-022-05022-3

**Published:** 2022-02-09

**Authors:** Ayuni Nur Apsari, Eko Sudoyo, Eka Mulya Alamsyah, Kenji Kobayashi, Takashi Tanaka

**Affiliations:** 1 The United Graduate School of Agricultural Science, Gifu University, 1-1 Yanagido, Gifu-shi, Gifu, 501-1193 Japan; 2 Research and Development Division, PT Sumber Graha Sejahtera, Sampoerna Strategic Square, North Tower, 21st Floor, Jl. Jend. Sudirman Kav. 45-46, Jakarta, 12930 Indonesia; 3 Post Harvest Technology Study Program, School of Life Sciences and Technology, Institut Teknologi Bandung, Jalan Ganesha No. 10, Bandung, 40132 Indonesia; 4 College of Agriculture, Academic Institute, Shizuoka University, Ohya 836, Suruga-ku, Shizuoka, 422-8529 Japan

**Keywords:** Composites, Soft materials

## Abstract

Recently, the plywood industry has been using low-molecule phenol (LMP) to enhance the dimensional stability of inferior-quality wood, along with copper naphthenate to increase veneer durability against staining fungi as a wood preservative. Non-destructive X-ray microtomography is a promising visualization method for reviewing the distribution of these materials. This study aimed to determine the applicability of X-ray microtomography for observing the distribution of LMP and Cu in two-and three-dimensional visualizations. The distribution mechanisms of these materials were investigated using X-ray images and image plot profiles. Six hardwood (wood from broad-leaved trees) and one softwood (wood from conifer trees) species were used for the experiments. An impregnation process was used to treat the wood samples with LMP, and copper naphthenate was added by dipping the wood in the compound for 1 s. A 10 mm^2^ sample of each wood species was scanned using X-ray microtomography, and the distribution of LMP and Cu was successfully visualized using X-ray microtomography with the same settings. The LMP was displayed approximately evenly throughout the veneer, whereas the copper naphthenate existed mainly on the veneer surface. The X-ray images successfully showed penetration at the microscopic scale.

## Introduction

The use of X-rays as a non-destructive technique to provide qualitative and quantitative analyses has been extensively researched. Many X-ray methods have been combined and developed to meet the need for evaluating treated wood. Wood is treated to increase its physical properties, and the quality of the treated wood can be evaluated by the spreading (penetration) of the treatment agent/reagent throughout the wood. The specific methods used to examine the micro-distribution of preservative materials using X-ray are energy dispersive analysis (EDX), X-ray fluorescence (XRF) microscopy, and X-ray micro-computed tomography (CT)^1^. The use of preservatives increases the density of wood; therefore, X-ray densitometry can be used to inspect the management of wood properties, including wood density after treatment^2^. In addition to clearly visualizing the reagent, the mass attenuation of the reagent is also scanned when using X-rays^3^. Organic materials are difficult to visualize because they contain the same chemical as that of the wood itself.

In recent years, the plywood industry has used various hardwood (wood from broad-leaved trees) and softwood (wood from conifer trees) species from industrial forests to be wood veneer (sheet) for their plywood. Those are inferior quality wood that has low density, many defects, weak, and often easily to get attack by fungi and termites^4^. The hardwood species used mostly a fast-growing species such as falcata wood (*Falcataria mollucana* (Miq.) Barneby & J.W. Grimes), Jabon wood (*Anthocephalus cadamba* (Roxb.) Miq), Surian wood (*Toona sinensis* (A.Juss.) M.Roem), Beechwood (*Gmelina arborea* Roxb.), Manglid wood (*Manglietia glauca* (Blume) Figlar & Noot.), and Rubber wood (*Hevea brasiliensis* (Willd. ex A. Juss.) Müll. Arg.). The softwood that commonly used mostly in Japan is Japanese cedar (*Cryptomeria japonica* (L. f.) D. Don).

Currently, low-molecule phenol (LMP) is often used to improve wood properties^5^ by enhancing its dimensional stability and biological features. Copper naphthenate has also been used to increase veneer durability, particularly for protection of the veneer surfaces against staining fungi as a wood preservative^6^. Appropriate instruments are needed for quality control of the LMP and copper naphthenate treated veneer to determine the distribution of those materials without wasting the tested material.

X-ray microtomography could be a promising method for non-destructive testing to measure and visualize the micro-distribution of LMP and copper naphthenate with high resolution and three-dimensional (3D) images without damaging the wood^7–9^. X-ray microtomography data can not only determine the distribution of the preservative reagent, but can also map the density of the treated wood and changes to the cell wall^10,11^. The penetration phenomena are affected by not only the atomic number of the reagent added to the wood, but also by its concentration, which can be visualized clearly using X-ray microtomography^12^.

Although X-ray microtomography is powerful for investigating the inner structure of polymer composite materials, the marginal difference in contrast between carbon-based materials such as petroleum-based plastics or organic materials is still an issue. Therefore, almost all previous studies have conducted the addition of contrast medium or chemical modification of materials before X-ray microtomography^9–11^. The addition or modification, however, is not suitable for industrial use, such as quality control.

More recently, a sub-microscale higher contrast CT apparatus was developed, which is suitable for carbon-based materials and thus can be used for the morphological evaluation of carbon fiber reinforced polymer (CFRP) materials^13^. Oishi and Tanaka have attempted to directly observe adhesive bondlines of plywood and glulam using six X-ray microtomography apparatuses and successfully visualized the wood adhesive at the bondlines without any addition or chemical modification^14^. In this study, however, the field of view (FOV) was at maximum 3.6 mm ϕ, which is not suitable for industrial applications.

Accordingly, the aim of this study was to determine the applicability of X-ray microtomography to distinguish nanatomical elements of wood (wood cell wall, lumen, among other variables), and the reagents (LMP and Cu) on LMP and copper naphthenate treated-wood using seven wood species. In relation to the quality assessment of the treated product, the penetration phenomena of LMP and Cu in the X-ray image and image plot profile were investigated. In this study, matters related to X-ray microtomography technique such as larger FOV, best image contrast quality for scientific morpho-anatomy analysis, simplicity of technique ensuring that the sample being scanned was without additives and chemical modifications, various wood species, and ingredients of the LMP and copper naphthenate material were checked.

## Methods

### Low-molecule phenol (LMP) and copper naphthenate treatment

**Figure 1:**
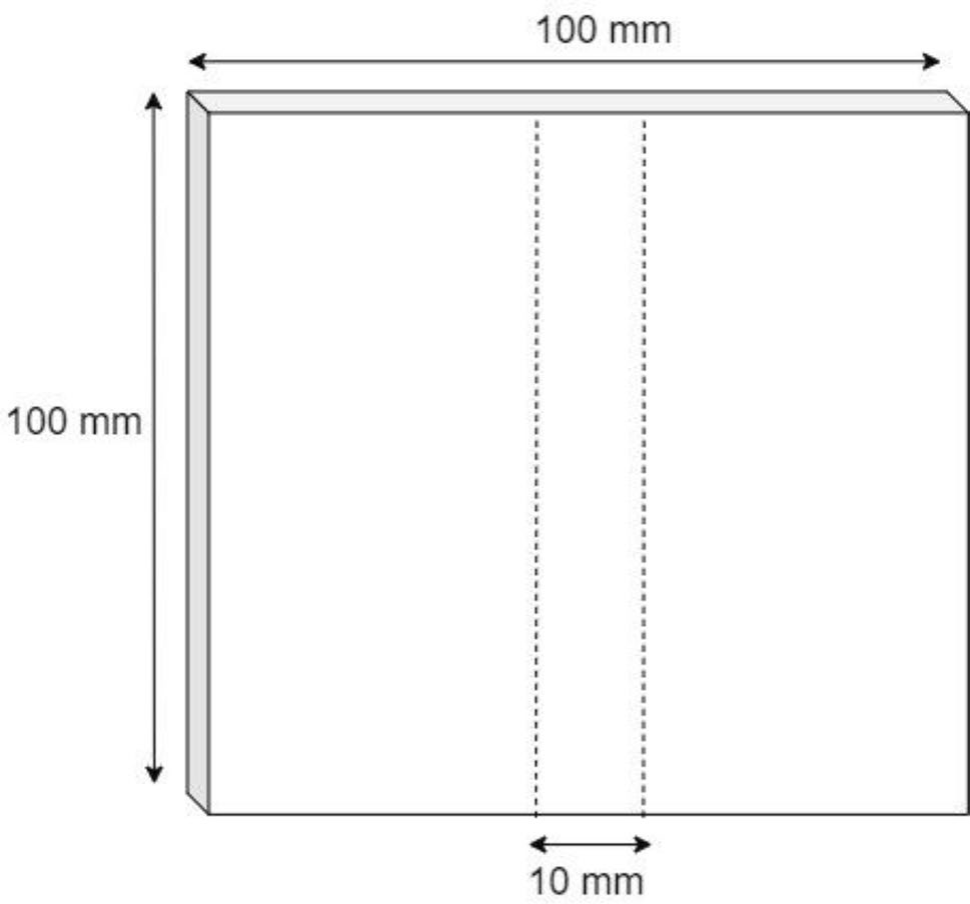
The veneer dimensions used for initial treatment (impregnation of low-molecule phenol and dipping of copper naphthenate), and X-ray scanning sample (dashed lines).

Hardwood and Japanese softwood veneers were used in this experiment with sample dimensions of 100 mm^2^ (Fig. 1).

Five rotary veneers were prepared for each wood species. One was used as a control (no checimal treatment to enhance the wood quality), three were impregnated with LMP, and one was dipped in copper naphthenate for 1 s. The physical properties of the reagents are listed in Table 1. The impregnation pressure of the LMP was maintained at 5 kg/cm^2^ for 30 min using a Krisbow KW20-425 pump. After treatment, all veneers were oven-dried at 103 °C for 15 h. The veneer density and weight percentage gain were calculated using Eqs. (1) and (2), respectively, and are presented in Table 2.

1 $$ {\varvec{\rho}} = \frac{{\user2{mass }\left( {\varvec{g}} \right)}}{{\user2{volume }\left( {{\varvec{cm}}^{3} } \right)}} $$

2 $$ {\varvec{weight}}\; {\varvec{percentage}}\;{\varvec{gain}}\; \left( \user2{\% } \right) = 100\user2{ \% } \times \frac{\left( {\varvec{final}}\; {\varvec{weight}}\; \left( {\varvec{g}} \right) - {\varvec{initial}}\; {\varvec{weight}}\;\left( {\varvec{g}} \right) \right)}{\left| {\varvec{initial}}\; {\varvec{weight}}\;\left( {\varvec{g}} \right) \right|} $$

**Table 1:** The physical properties of low-molecule phenol (LMP) and copper naphthenate.

LMP		Copper naphthenate	
Specifications	Value	Specifications	Value
pH	12	Nonvolatile (%)	80
Viscosity (P)	3.5	Viscosity (Cps)	285
Specific gravity	1.14	Specific gravity	0.998
Solid content (%)	27.11	Metal content (%)	8

**Table 2:** The density and weight percentage gain of the low-molecule phenol and copper naphthenate (Cu) treated veneers and untreated veneer (control).

Species name	Treatment	Oven dried density (g/cm^3^)	Density after treatment (g/cm^3^)	Weight percentage gain (%)
Japanese cedar heartwood *Cryptomeria japonica* (L. f.) D. Don	Control	0.27	–	–
	LMP	0.26	0.43	63.16
	Cu	0.27	0.28	2.56
Falcata wood from Sumedang city *Falcataria moluccana* (Miq.) Barneby & J.W. Grimes	Control	0.27	–	–
	LMP	0.25	0.38	51.40
	Cu	0.25	0.25	1.69
Falcata wood from Jambi city *Falcataria moluccana* (Miq.) Barneby & J.W. Grimes	Control	0.25	–	–
	LMP	0.28	0.39	37.83
	Cu	0.22	0.23	2.61
Rubber wood from Sumedang city *Hevea brasiliensis* (Willd. ex A. Juss.) Müll. Arg	Control	0.64	–	–
	LMP	0.60	0.75	25.75
	Cu	0.63	0.64	0.47
Rubber wood from Jambi city *Hevea brasiliensis* (Willd. ex A. Juss.) Müll. Arg	Control	0.68	–	–
	LMP	0.70	0.85	21.14
	Cu	0.64	0.65	0.69
Jabon wood from Sumedang city *Anthocephalus cadamba* (Roxb.) Miq	Control	0.29	–	–
	LMP	0.35	0.50	43.99
	Cu	0.29	0.29	1.63
Surian wood *Toona sinensis* (A.Juss.) M.Roem	Control	0.41	–	–
	LMP	0.43	0.50	16.53
	Cu	0.42	0.42	0.71
Beechwood *Gmelina arborea* Roxb	Control	0.42	–	–
	LMP	0.50	0.58	16.22
	Cu	0.42	0.42	0.00
Manglid wood *Manglietia glauca* (Blume) Figlar & Noot	Control	0.62	–	–
	LMP	0.51	0.63	23.56
	Cu	0.58	0.58	0.72

### Sample preparation for X-ray microtomography

**Figure 2:**
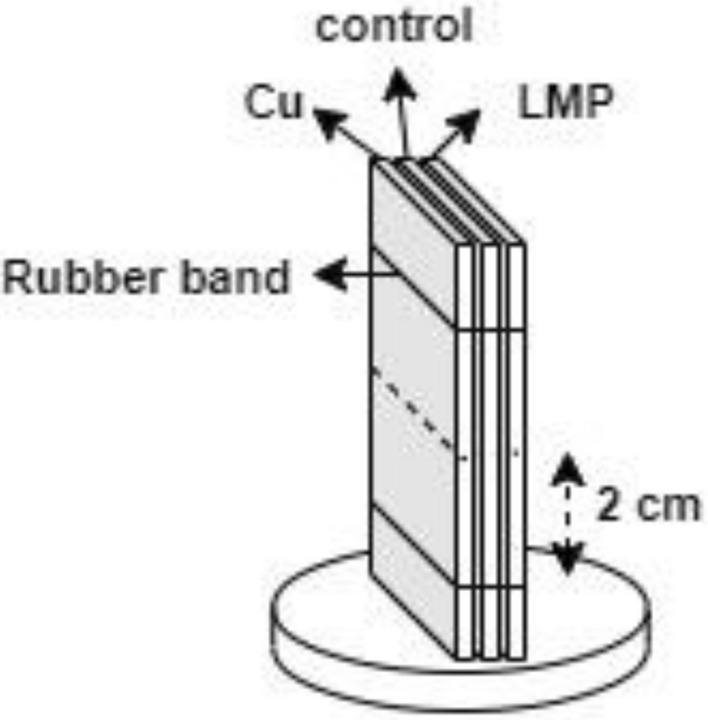
X-ray sample arrangement.

The X-ray sample tests were prepared with dimensions of 10 mm^2^ based on the FOV of the apparatus (Lens L4320, FOV, 14 mm × 10 mm, 4.4 µm/pixel). The X-ray samples were ordered from the observable window of the apparatus (front) to the back as copper naphthenate treated-veneer, untreated-veneer (control), and LMP-treated veneer (Fig. 2). The three samples were then tied together with a rubber band. A focus line was drawn 2 cm from the bottom rubber band to indicate the focus point of the scanning object.

### X-ray microtomography scanning

**Figure 3:**
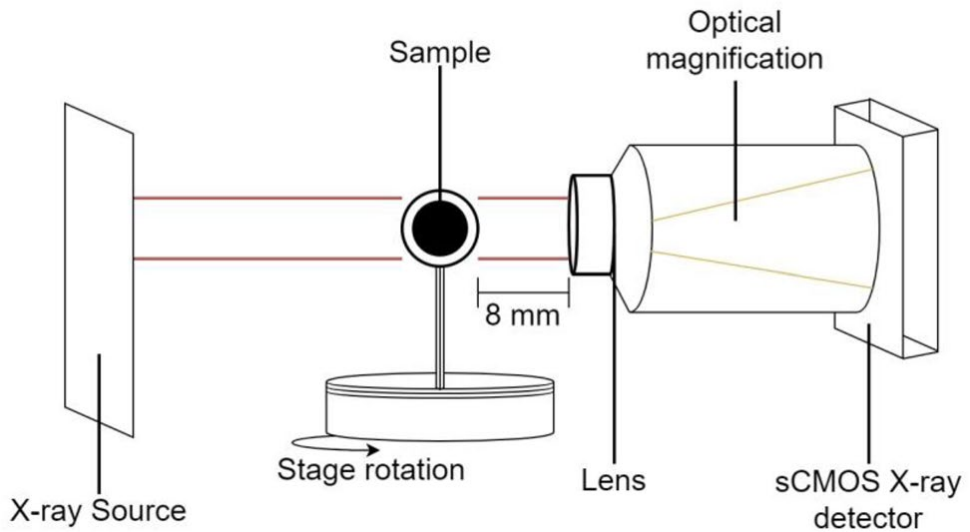
Schematic of the X-ray microtomography apparatus.

X-ray samples of all species were scanned using an X-ray microtomography apparatus (Nano 3DX, Rigaku Corporation, Tokyo, Japan) with a 50 kV tube voltage, 24 mA tube current, sCMOS X-ray detector equipped with L4320 lens, and a molybdenum X-ray target (Fig. 3). The acquisition settings were binning 2 (resulting in 8.800 µm/px), 8 s exposure time, angular step at 0.225° (with 800 projections equaling 180° ÷ 800 = 0.225 angular steps), and the distance between the sample and lens was 8 mm, which was reported as an appropriate setting for wood-based material observation^15^. After scanning, we auto-centered the reconstruction image because the sample shifted during the rotation of the X-ray scanning process.

### X-ray image analysis

The X-ray image stack obtained from each wood species that was scanned after the reconstruction process had 1236 images with a resolution of 1644 × 1644 pixels. These were analyzed using ImageJ 1.48v software to obtain the gray value plot profile, which represents the material distribution in the wood. Further investigation of the distribution of LMP and Cu in the treated veneer compared with the untreated veneer (control) was performed on the 3D X-ray image using VGstudio software.

## Results and discussion

### Density and weight percentage gain

The density change after treatment with LMP increased for all species; however, the density of seven species after copper naphthenate treatment showed no increase. The weight percentage gain of LMP-treated veneer was greater than that of the copper naphthenate treated veneer (Table 2). A possible reason for these differences is the application method used. The impregnation process (vacuum pressure) changed the air inside the wood cell to LMP^16^, whereas the dipping process for copper naphthenate did not allow the Cu to completely fill the wood; therefore, there was no significant weight gain.

### 2D X-ray image visualization

**Figure 4:**
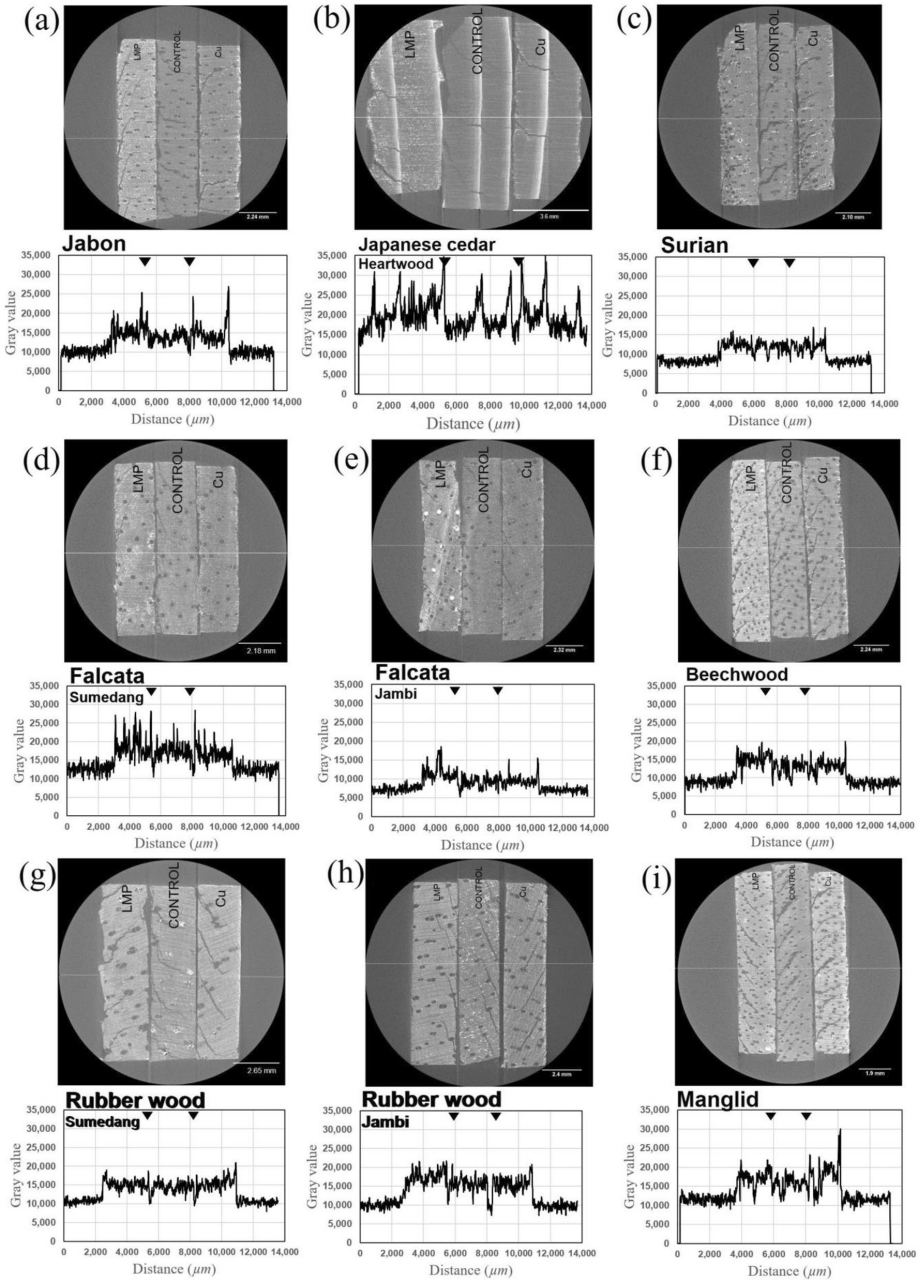
The 2D X-ray visualization of various woods. (**a**) Jabon wood from Sumedang city, (**b**) Japanese cedar heartwood veneer, (**c**) Surian wood, (**d**) Falcata wood from Sumedang city, (**e**) Falcata wood from Jambi city, (**f**) Beechwood, (**g**) Rubber wood from Sumedang city, (**h**) Rubber wood from Jambi city, and (**i**) Manglid wood, including the image gray value of the yellow middle line.

The LMP and Cu 2D visualization and gray value plot profile distribution are shown in Fig. 4. The distribution of LMP and Cu in the veneer was successfully observed using X-ray microtomography. The LMP was distributed uniformly to a certain degree throughout the veneer, whereas copper naphthenate was mainly present on the surface. The LMP-treated and copper naphthenate-treated samples appeared brighter with higher gray content than the untreated control. Cu was observed owing to its high atomic number. In contrast, although LMP has the same chemical structure as wood, it was still differentiated from the control because of its high concentration. The average gray value plot profile (Table 3) shows that the distribution of LMP is consistently higher than that of the control. It also reveals that the gray value of the surface of the copper naphthenate samples is higher than that of the internal section. The base gray value plot profile was similar for all species. This indicates that X-ray microtomography is suitable for identifying the variation in wood species using the same X-ray settings, and this was indicated for a majority of the wood species. The morpho-anatomy of each wood species treated with LMP and copper naphthenate, and the untreated veneer (control) was also successfully visualized in the X-ray image.

**Table 3:** Average gray value differences of all hardwood, softwood, and non-wood species.

Species name	Average gray value		
	Control	LMP	Cu
Japanese cedar heartwood	18,555.73	21,443.93	19,785.43
Falcata wood from Sumedang city	17,436.26	18,902.41	16,762.45
Falcata wood from Jambi city	9,178.57	11,731.48	9,478.11
Rubber wood from Sumedang city	14,507.30	15,226.38	15,520.58
Rubber wood from Jambi city	15,749.64	17,488.68	15,655.63
Jabon wood from Sumedang city	13,513.15	15,692.20	14,775.80
Surian wood	11,934.19	12,250.29	12,082.27
Beechwood	12,807.04	15,215.74	13,149.06
Manglid wood	15,601.04	17,252.75	18,094.21

Jabon wood and Beechwood (Fig. 4a, f) X-ray images show the differences between the treated and untreated samples. The image of the LMP-treated veneer was the brightest, which is supported by the plot profile that revealed the most peaks for these woods, thereby resulting in a high average gray value (Table 3).

The anatomy of Japanese cedar wood is that of thick latewood^17^. The distribution of LMP in the latewood was unclear based on the X-ray images (Fig. 4b), although the plot profile showed that the peak of the latewood portion of the treated veneer was higher than that of the control.

The Surian wood X-ray image had many objects in the image (Fig. 4c) because of the octahedral silica that lines the rays^18^; therefore, a region dominated by gray color was expected on the X-ray image near the rays that displayed a silica octahedron.

In the Falcata wood (Fig. 4d, e), a bright point is concentrated in the wood vessel of the LMP-treated veneer, which indicates the presence of the reagent. Regarding the anatomy of Falcata wood, the pore (trachea) is clearly visible and can be easily distinguished in the X-ray image. The main observation with this wood was the reduction in the size of the treated veneer compared with the untreated veneer (control), which was related to the LMP infusion (Fig. 4e).

Rubber wood from community and industrial forests differed in their anatomy, and this was successfully observed in the X-ray microtomography images, as shown in Fig. 4g, h. The evidence of tyloses that are common in rubber wood vessels can be seen clearly on the untreated veneer (control) of the sample from the community forest (Fig. 4g), whereas the existence of prismatic crystals can be observed in the control of samples from the industrial forest (Fig. 4h).

Based on the average gray value, rubber wood from the community forest and Manglid wood treated with copper naphthenate showed higher gray values than wood treated with LMP (Table 3). The plot profile also revealed the highest peak on the surface of the copper naphthenate treated-veneer (Fig. 4g, i).

### Preliminary 3D X-ray image analysis

The use of 3D image analysis is a convenient way to track the LMP and Cu inside the treated wood, especially in the vessel sections (transverse, radial, and tangential sections). The preliminary analysis can enable identification of the reagent by combining the image stacks to form a 3D visual and by providing a review of the transverse, radial, and tangential sections.

**Figure 5:**
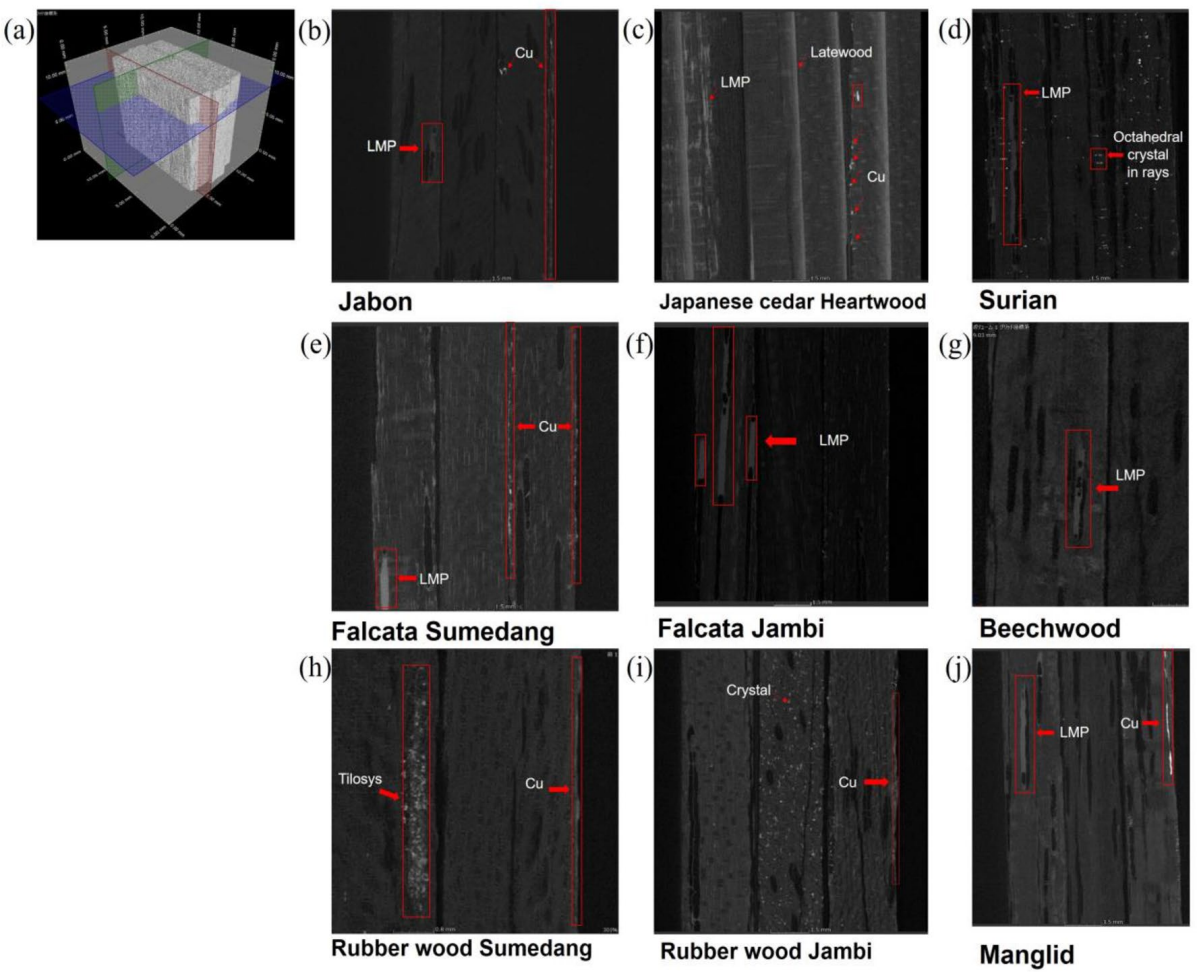
A 3D X-ray observation of the distribution of LMP and Cu. (**a**) A 3D image stack of the treated-veneer by LMP and copper naphthenate, and the untreated veneer; softwood and hardwood 2D representative images of a radial section were obtained from the green cut section of the 3D image, (**b**) Jabon wood from Sumedang city, (**c**) Japanese cedar heartwood veneer, (**d**) Surian wood, (**e**) Falcata wood from Sumedang city, (**f**) Falcata wood from Jambi city, (**g**) Beechwood, (**h**) Rubber wood from Sumedang city, (**i**) Rubber wood from Jambi city, and (**j**) Manglid wood.

Figure 5 shows one of the 3D images obtained using Manglid wood. The blue, green, and red layers indicate the active 2D image on the transverse section, the radial section, and the tangential section, respectively. The LMP and Cu in the vessel can be verified based on the intersection of the three sections. This discovery can induce further segmentation steps and calculations that clarify the penetration phenomena and quantitative analyses.

## Conclusions

X-ray microtomography can be used to visualize LMP and Cu in treated wood. The same X-ray microtomography setting is relevant for observing the LMP and Cu in species with different types of wood (hardwood and softwood). The X-ray images show unique phenomena of the treated wood based on the morpho-anatomy of each wood species. Size changes were observed in the treated wood samples, and the tyloses, octahedral crystal, and silica in certain varieties, and the LMP and Cu materials concentrated in the wood vessel were successfully visualized in 2D and 3D images. The average gray value from the plot profile can quantitatively describe the distribution of LMP and Cu in the treated wood to assess the quality related to the uniformity and heterogeneity of the distribution. The visualization is more focused on smaller samples, Mo X-ray targets, and smaller FOV lenses with high binning settings. The results described here were provided by the non-destructive technique of visualization using X-ray microtomography, which is a powerful and promising method to observe woody materials and reagents in treated wood for quality control in industrial applications.
